# Suicide rates in the UK Armed Forces, compared with the general workforce and merchant shipping during peacetime years since 1900

**DOI:** 10.1136/military-2022-002309

**Published:** 2023-04-07

**Authors:** Stephen E Roberts, A John, T Carter, J G Williams

**Affiliations:** 1 Medical School, Swansea University, Swansea, UK; 2 Norwegian Centre for Maritime and Diving Medicine, Haukeland Universitetssjukehus, Bergen, Norway

**Keywords:** Suicide & self-harm, MENTAL HEALTH, OCCUPATIONAL & INDUSTRIAL MEDICINE, PREVENTIVE MEDICINE

## Abstract

**ABSTRACT:**

**Introduction:**

The main objective was to compare suicide rates and their trends across the three UK Armed forces (Royal Navy, Army and Royal Air Force) from 1900 to 2020. Further objectives were to compare suicide rates with those in the corresponding general population and in UK merchant shipping and to discuss preventative measures.

**Methods:**

Examination of annual mortality reports and returns, death inquiry files and official statistics. The main outcome measure was the suicide rate per 100 000 population employed.

**Results:**

Since 1990, there have been significant reductions in suicide rates in each of the Armed Forces, although a non-significant increase in the Army since 2010. Compared with the corresponding general population, during the most recent decade from 2010 up to 2020, suicide rates were 73% lower in the Royal Air Force, 56% lower in the Royal Navy and 43% lower in the Army. Suicide rates have been significantly decreased in the Royal Air Force since the 1950s, in the Royal Navy since the 1970s and in the Army since the 1980s (comparisons for the Royal Navy and the Army were not available from the late 1940s to the 1960s).

During the earliest decades from 1900 to the 1930s, suicide rates in the Armed Forces were mostly quite similar or moderately increased compared with the general population, but far lower than in merchant shipping. Following legislative changes in the last 30 years, suicide rates through poisoning by gases and through firearms or explosives have fallen sharply.

**Conclusions:**

The study shows that suicide rates in the Armed Forces have been lower than in the general population over many decades. The sharp reductions in suicide rates over the last 30 years suggest the effectiveness of recent preventative measures, including reductions in access to a method of suicide and well-being initiatives.

**WHAT IS ALREADY KNOWN ON THIS TOPIC**

- Long-term trends in suicide rates have not previously been collated and compared for the three UK Armed Forces (during service), merchant shipping and the general population.

**WHAT THIS STUDY ADDS**

- Since 1990, there have been significant reductions in suicide rates in each of the Armed Forces, with a minor non-significant increase in the Army since 2010.
- Compared with the general population, suicide rates have been significantly lower in the Royal Air Force since the 1950s, the Royal Navy since the 1970s and the Army since the 1980s.
- The sharp reduction in suicide rates in the Armed Forces over the last 30 years suggest the effectiveness of preventative measures and well-being initiatives.

**HOW THIS STUDY MIGHT AFFECT RESEARCH, PRACTICE OR POLICY**

- Current policies on identification of mental ill-health and interventions need to be maintained, along with monitoring of any increases in suicides in the Army and in service personnel discharged.

## Introduction

 Comparative long-term trends in suicide rates in the Armed Forces (Royal Navy, Army and Royal Air Force), the general population and merchant shipping have not been collated and reported previously. Both the military and merchant seafaring have been reported as occupations with high suicide rates in the UK and internationally.^1^,^13^ Recent studies from the UK, however, have reported reduced suicide rates in the military when compared with general populations.^14 15^ The Armed Forces and merchant seafaring are similar in that most of those employed are typically in service or on contracts of employment which often involve long periods of time spent on overseas deployment or at sea; hence the inclusion of merchant shipping as an additional occupation which shares some of the features of the primary study populations.

Unlike almost all land-based occupations, fatalities in the British Armed Forces and merchant shipping during service have not usually been administered in the same way as the rest of the population. They are not normally registered with local registrars of deaths nor included in central mortality returns. Instead, they have been administered and registered separately with, respectively, the Ministry of Defence and the Registry of Shipping and Seamen. Suicide rates for other occupations such as farmers, veterinarians and doctors are well documented from central mortality sources and from previous publications.^16^,^18^

The main objective of this study was to compare suicide rates and their trends across the UK Armed Forces since 1900. Further objectives were to compare suicide rates and their trends with those in the corresponding general population and in UK merchant shipping, to report on the method of suicide and to discuss preventative measures for suicides.

## Method

This study covered the 121-year period from 1900 to 2020, excluding the two world war years from 1915 to 1918 and 1940 to 1945 when information on fatalities was largely unavailable for the study occupations. The main information sources used for identifying suicides and the populations employed in each of the three Armed Forces, British merchant shipping and in the general population are detailed in online supplemental appendix. These include annual death returns and historical mortality reports from the Ministry of Defence, Medical Director of the General Admiralty, Air Ministry, Board of Trade, Ministry of Transport, Department of Trade and Industry, the Marine Accident Investigation Branch, and the Registry of Shipping and Seamen. Additionally, for merchant shipping, death inquiry files were accessed. These were sourced at or from specialist archives, libraries and museums across England and Wales; including the Ministry of Defence Burnett Library at Whittington barracks, Lichfield; the Institute of Naval Medicine Historic Library, Gosport; the Air Historical Branch museum, London; the Wellcome Trust library, London; Swansea University library; the Registry of Shipping and Seamen, Cardiff; and the Marine Accident Investigation Branch, Southampton. Additional statistical data on suicides in the Armed Forces were provided by Defence Statistics Health.

In some years, annual mortality reports or returns were not released or could not be located from the study information sources. In other years, suicides were included under an ‘injury and poisoning’ categorisation and could not be distinguished from accidents, war casualties, homicides and other fatalities from unnatural causes. Consequently, these years were excluded from the analysis for the respective occupations. For the four occupations studied, annual suicide rates were available as follows: for the Royal Navy (76), Army (69), Royal Air Force (81 years) and merchant shipping (99). Details of the years with missing suicide data are provided in the online supplemental appendix.

The suicides included in this study were those that occurred among men and women in regular service for the Armed Forces and, for merchant shipping, when signed on UK-registered merchant ships. For the Armed Forces, the study excluded suicides among those who had been discharged from active service or were in reserve forces. The study included deaths classified as ‘open verdicts’ through coroners’ inquisitions along with ‘suicide verdicts’. For merchant shipping, the study excluded suicides that arose when personnel were signed off ships, on shore leave or had been discharged ashore through sickness. For the general population, numbers of suicides and resident populations were obtained from the Office of National Statistics.^19^,^21^ The International Classification of Diseases codes for suicides (from version 1 in 1901 to version 10 in 2020) are included in the online supplemental appendix.

The main study outcome measure is the suicide rate per 100 000 person-years employed in the study occupations and in the general population. Methods of analysis include trends in suicide rates over the 121-year study duration, which were smoothed using moving averages. Relative risks of suicide rates in the study occupations were compared with those in the general British male workforce aged 15–44 years. Logistic regression models were used to assess trends over time in suicide rates overall, for the most common methods of suicide used, and during time periods before and after suicide national prevention legislation was introduced. The χ^2^ test was used to assess differences between the methods of suicide used in the Armed Forces and merchant shipping. Statistical significance was measured at the conventional 5% level.

## Results

For the years since 1900 with available data, there were 566 suicides in the Royal Navy (over 76 years), 473 suicides recorded for Royal Air Force personnel (81 years), 1489 in the Army (69 years), compared with 4062 in UK merchant shipping (99 years). A total of 41 627 suicides were identified for the corresponding general British male population aged 15–44 years. Over the entire study period since 1900, overall suicide rates were lowest in the Royal Air Force (7.8 per 100 000 population), followed by the Royal Navy (9.6 per 100 000), the Army (13.6 per 100 000) and merchant shipping (23.4 per 100 000).

During the recent 21 years from 2000 to 2020, suicide rates were 7.2 in the Royal Navy per 100 000 population, 10.1 per 100 000 in the Army, 5.0 per 100 000 in the Royal Air Force, 8.3 per 100 000 in the Armed Forces overall and 3.2 per 100 000 in merchant shipping. In the corresponding general population aged 15–44 years, the suicide rate during this period was 16.7 per 100 000.

### Trends in suicide rates over time

**Figure 1:**
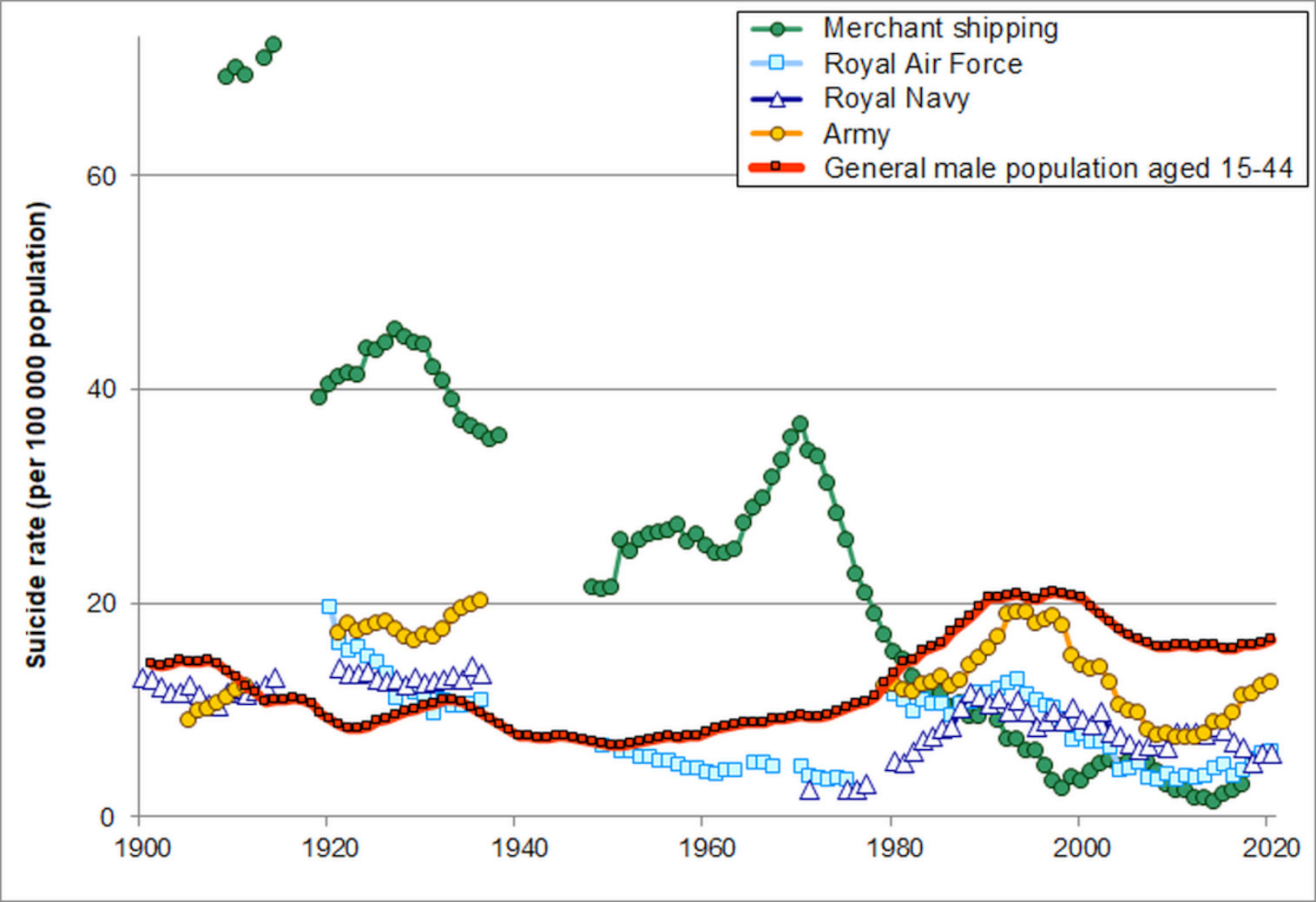
Trends in suicide rates for the UK Armed Forces, merchant shipping and the general male population aged 15–44 years since 1900.

**Figure 2:**
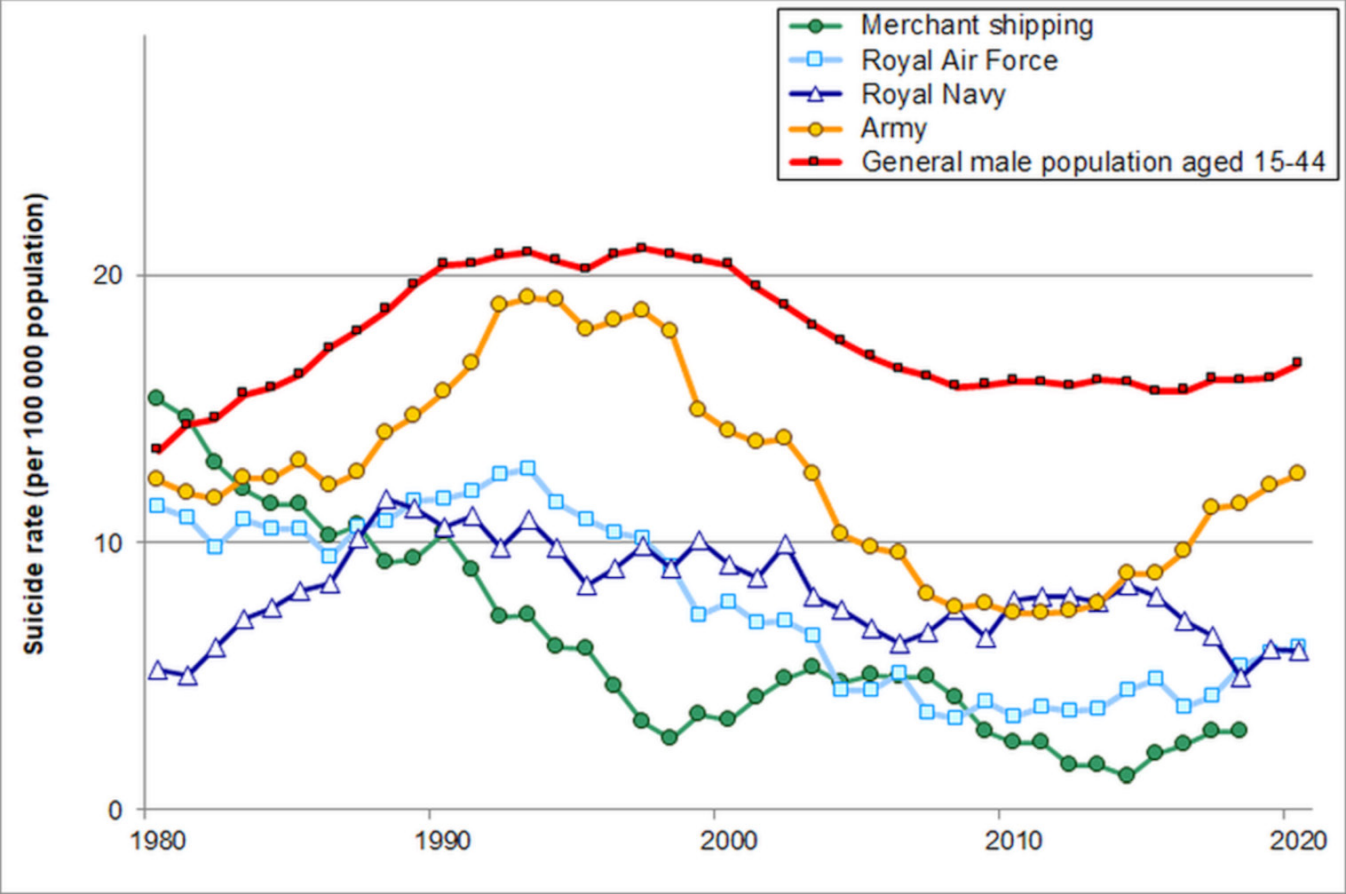
Trends in suicide rates for the UK Armed Forces, merchant shipping and the general male population aged 15–44 years since 1980.

Figure 1 shows that suicide rates were far lower in each of the Armed Forces than in UK merchant shipping up to the 1970s. Suicide rates have been quite stable and moderate in the Armed Forces over the twentieth century (5–20 per 100 000 during almost all years since 1900). Since 1990 there have been significant reductions in suicide rates in the Royal Air Force (% annual reduction=5.3%; 95% CI 3.2% to 7.3%) and in the Royal Navy (2.6%; 95% CI 0.5% to 4.6%; figure 2). In the Army there was a significant fall from 1990 to 2010 (5.1%; 95% CI 3.4% to 6.7%) but a non-significant increase since 2010 (3.7%; 95% CI −3.4% to 11.3%).

Much higher suicide rates in merchant shipping of approximately 70 per 100 000 population before 1914 fell sharply over time, to 35–45 per 100 000 in the 1920s and 1930s, 20–40 from the late 1940s to the 1970s, with a sharp decline to <10 since the 1990s.

### Comparison of suicide rates with the general population

Compared with the corresponding general population, over the entire study period since 1900, suicide rates have been significantly lower in the Royal Air Force and Royal Navy (relative risks=0.53 and 0.63, respectively; table 1). Suicide rates have been slightly higher in the Army (1.08) and much higher in merchant shipping (2.28).

**Table 1:** Relative risks of suicide in the UK Armed Forces and in UK merchant shipping compared with the general male population aged 15–44 years since 1900

Time period	Relative risk of suicide in each occupation compared with the general population							
	Royal Navy		Army		Royal Air Force		Merchant shipping	
	Relative risk	(No. of risk suicides)	Relative risk	(No. of risk suicides)	Relative risk	(No. of risk suicides)	Relative risk	(No. of risk suicides)
1900–1914	0.90	(202)	0.79 *	(172)	†		4.98‡	(827)
1919–1929	1.50‡	(111)	1.88 ‡	(315)	1.61‡	(34)	4.29‡	(1143)
1930–1939	1.33‡	(80)	1.86 ‡	(255)	1.01	(21)	3.36‡	(690)
1946–1959	n/a		n/a		0.81*	(129)	2.33‡	(596)
1960–1969	n/a		n/a		0.49*	(41)	3.01‡	(429)
1970–1979	0.26*	(8)	1.22 ‡	(104)	0.35*	(24)	2.72‡	(281)
1980–1989	0.46*	(52)	0.85 *	(205)	0.61*	(83)	0.37*	(60)
1990–1999	0.50*	(57)	0.62 *	(221)	0.56*	(84)	0.24*	(13)
2000–2009	0.42*	(29)	0.62 *	(124)	0.29*	(29)	0.29*	(7)
2010–2020	0.44*	(27)	0.57 *	(93)	0.27*	(19)	0.10*	(6)
1900–2020	0.63*	(566)	1.08 ‡	(1489)	0.53*	(473)	2.28‡	(4062)

Details of years with missing annual mortality returns for each occupation are provided in the online supplemental appendixAppendix.

* Denotes a significantly (pp<0.05) reduced relative risk of suicide compared with the corresponding general population (of malesmen aged 15–44 years).

† The Royal Air Force was not founded until 1918.

‡ Denotes a significantly (pp<0.05) increased relative risk of suicide compared with the corresponding general population (of malesmen aged 15–44 years).

n/anot available

Compared with the general population, suicide rates in the Royal Air Force have been significantly lower since the 1950s (table 1). They have also been significantly lower in the Royal Navy since the 1970s and in the Army since the 1980s (and were not available for both services from the late 1940s to the 1960s). During the early decades from 1900 to the 1930s, suicide rates in the Armed Forces were mostly quite similar or moderately increased, when compared with the general population, with largest increased rates for the Army (relative risk=1.88 in the 1920s and 1.86 in the 1930s). In merchant shipping, suicide rates were significantly much higher than in each of the Armed Forces and the general population from the 1900s (relative risk=4.98 compared with the general population) up to the 1970s (relative risk=2.72) and significantly reduced since then.

### Method of suicide

There have been major differences in the method of suicide in the (male) Armed Forces compared with merchant shipping (p<0.001) in recent decades. Of 873 suicides in the male Armed Forces since 1984, the method of suicide was hanging, strangulation or suffocation in 39% of cases, poisoning by gases and vapours (21%), firearms and explosives (19%), poisoning by liquid and solid substances (6.2%), jumping from heights (3.3%), road traffic (2.9%) or railway-related suicides (2.7%), drowning (1.3%) and other or unspecified means (3.3%). Of 136 suicides in merchant shipping since 1984, 89% were due to drowning (mostly by jumping overboard), 8.1% were by hanging, strangulation and suffocation, and 2.9% were from other causes.

In the Armed Forces between 1984 and 2020 (for the three most common methods of suicide), there were significant reductions in suicide rates for firearms and explosives (mean annual reduction=3.1%; 95% CI 1.6% to 4.6%) and for poisoning by gases and vapours (8.8%; 95% CI 7.0% to 10.6%) but a modest significant increase for hanging, strangulation and suffocation (1.8%; 95% CI 0.1% to 2.8%).

For suicides through firearms and explosives and also for poisoning by vapours and gases, there were significant increases in suicide rates from 1984 to 1993, respectively (9.7% mean annual increase; 95% CI 1.1% to 18.9% and 6.0%; 95% CI 0.0% to 12.5%, respectively) but subsequent significant reductions from 1994 to 2020, respectively (8.0%; 95% CI 4.9% to 11.0% and 16.5%; 95% CI 11.1% to 21.6%). For hanging, strangulation and suffocation, there was no significant trend pre or post 1994.

## Discussion

The study covers more than 2500 suicides in the Armed Forces and more than 4000 in UK merchant shipping, based on mortality reports and annual death returns, official statistics and merchant shipping death inquiry files. These were accessed through specialised archives, libraries and museums across England and Wales (at Gosport, Lichfield, London, Southampton, Swansea and Cardiff).

Since 1990, there have been significant reductions in suicide rates in each of the Armed Forces, although a non-significant increase in the Army in the most recent years since 2010. The latter could reflect a lack of statistical power or possible delayed responses to trauma, following recent conflicts, as well as possible increases over time in people recruited with childhood adversities.^22 23^

The overall decreasing trends in suicide rates for the three UK Armed Forces combined contrast with reports of increasing suicide rates in the US military from 2001 to 2008,^24^ and a stable trend in the German military from 2010 to 2016.^25^ The low overall suicide rate of 8 per 100 000 in the UK (male) Armed Forces since 2000 is lower than 19 per 100 000 in the French military from 2006 to 2010,^26^ 13 in the German military from 2010 to 2016,^25^ and approximately 15 in the US military from 2001 to 2008.^24^ This may reflect variation in recruitment patterns, differing combat deployments and uptake of preventative measures.

Of the three Armed Forces, suicide rates have usually been higher in the Army than in the Royal Navy and Royal Air Force. This may reflect differences in patterns of recruitment and socioeconomic status across the three services, differences in duties and management styles, and variation in access to a means of suicide and programmes directed at well-being and mental health.

A Ministry of Defence review of suicides across the UK tri-services from 1984 to 2020 reported highest age-specific suicide rates (of >25 per 100 000 employed) among men aged <20 years during the late 1980s and early 1990s and among those aged 40–44 years during the mid-1980s.^15^ A study of the US Army from 1998 to 2011, similarly reported highest suicide rates among men aged 20–29 years.^27^ The recent rise in male Armed Forces suicides was highest among those aged under 24 years and over 40 years, and an increase in suicide rates in these age groups was also seen in the general population.^15^

During earlier study years around 1910, the suicide rate in UK merchant shipping was about five times higher than in the Armed Forces and in the corresponding general population. These elevated suicide rates are broadly concordant with previous studies of Swedish merchant shipping that reported suicide relative risks of 20 in 1907,^8^ and 4.4 from 1945 to 1954,^10^ compared with Swedish general populations.

The high suicide rate in British merchant shipping around 1910 occurred when return oceanic voyages of more than 9 months were still common. The suicide rate fell through to the 1960s, with shorter voyage times, reduced occupational hazards and improvements in living and working conditions, welfare and recruitment practices. A sharp reduction over the last 50 years has coincided with a decline in oceanic cargo shipping as overseas trading and logistics have changed over time.

Compared with the corresponding general population, suicide rates have been significantly lower in the Armed Forces throughout almost all decades since the 1950s with available suicide information. This significant decrease has widened in recent decades, further suggesting the effectiveness of recent preventative measures, reductions in access to methods of suicide, along with well-being initiatives.

There was large variation in the method of suicide used in the male Armed Forces and in merchant shipping, which partly reflects that suicides when temporarily signed-off merchant ships are not included. During recent decades, hanging, strangulation and suffocation, poisoning by gases and vapours, and the use of firearms and explosives accounted for most cases in the Armed Forces, whereas drowning was the main method used in merchant shipping. This suggests that the likelihood of suicide is linked strongly to the ease of access to an effective method, both in the Armed Forces, and in merchant shipping.

The use of ‘poisoning by gases or vapours’ was the most common method of suicide in the male Armed Forces until UK legislation was changed in the mid-1990s to fit catalytic converters to vehicles, following which there was a steep decline in the suicide rate.^15^ During earlier decades, the suicide rate in the corresponding general population has been linked partly with coal gas poisoning,^28^ followed, in recent decades, by an increase in suicide by hanging.^29^ The change in policy in the mid-1990s that restricted access to weapons in the Army has coincided with a significant fall in the suicide rate by ‘firearms and explosives’. Legislation in 1998 that restricted the amount of ‘over the counter’ painkillers that could be purchased, has also helped reduce suicides through poisoning by solid substances.^15^ The suicide rate by hanging rose in the male Armed Forces following legislative restrictions in access to gases, vapours and firearms in 1993, and remains the most common method in the Armed Forces.^15^ Meanwhile, suicide rates by ‘gases and vapours’ and ‘firearms and explosives’ for the latest 5-year period 2016–2020 were at their lowest since 1984.^15^

A modest increased rate of suicide has been reported following discharge from the military in the UK during the years from 1996 to 2005.^30^ This suggests that risks of suicide in the Armed Forces, while reduced when in service increase following discharge. This may be evidence of factors such as delayed responses to traumatic events, selective discharge of young people with health problems, psychological distress among early service leavers with a history of childhood adversities or the loss of the camaraderie of military service.

### Limitations

Limitations are that the annual mortality returns were not released or could not be located for every year since 1900 and, in some years, they did not distinguish suicides from other deaths through injury or poisoning. Inclusion criteria for suicides differ slightly between the Armed Forces and merchant shipping. For the Armed Forces, it includes all suicides when in service, whereas in merchant shipping it covers only those that occur at sea or during each time-limited contract of employment. This would result in more complete suicide coverage for the Armed Forces, whereby suicides in the merchant navy, when temporarily signed off ships or on (short-term) shore leave, would not usually be included. The Armed Forces and merchant seafarers were drawn largely from England, Scotland, Wales and Northern Ireland, whereas the general population comparison covers only England and Wales. As there is a population difference of 11% between the UK and England & Wales, as of 2020,^15^ this should not affect the suicide rate comparisons substantially. The comparator general population is based on men aged 15–44 years. Although not an ideal comparison, this age-sex demographic category would probably most resemble those in the Armed Forces and in merchant shipping over the course of the study since 1900.

The inclusion, formally, of deaths from undetermined intent (or ‘open verdicts’ at coroners’ inquests)—along with suicides—began in 1968, with the introduction of the eighth revision of the International Classification of Diseases. However, this does not appear to have affected the trends in suicide rates substantially. The main study information sources for the Armed Forces do not provide information on combat deployments or other aspects such as the nature of duties, the age of the deceased and history of adverse childhood events, that may provide some explanation for suicide rate trends and patterns.

## Conclusions

Suicides rates in the three Armed Forces have been moderate and quite stable over the twentieth century. Since 1990, there have been significant reductions in suicide rates in each, although a slight non-significant increase in the Army in recent years since 2010. Compared with the general population, over time, suicide rates have significantly decreased in the Royal Air Force since the 1950s, in the Royal Navy since the 1970s and in the Army since the 1980s. Current policies on the identification of mental ill-health and interventions need to be maintained, as well as monitoring of any increases in suicides in the Army and in service personnel discharged.

## supplementary material

10.1136/military-2022-002309 online supplemental appendix 1

## Data Availability

Data are available upon reasonable request.
