# Supplementary material for: Suicide rates in the UK Armed Forces, compared with the general workforce and merchant shipping during peacetime years since 1900
Source: BMJ Mil Health. 2023 Apr 7;170(e2):e002309. doi: 10.1136/military-2022-002309 (PMC11671895; doi:10.1136/military-2022-002309)
Supplement: online supplemental appendix 1 [file military-170-e2-s001.pdf]

Appendix

1. Study information sources

Armed forces:

Ministry of Defence. Deaths in the UK regular armed forces [annual returns, 2001-2020]. London: Ministry of Defence, 2002-2021.

Medical Director – General Admiralty / Ministry of Defence. Reports on The Health of the Navy [annual returns, 1900-1914, 1921-1936, 1971, 1975-1977, 1980-2000]. London: HMSO, 1901-1915, 1922-1937, 1972, 1976-1978, 1981-2001.

Director General, Army Medical Services / Ministry of Defence. Reports on the Health of the Army [annual returns, 1905-1911, 1921-1936, 1975-2000]. London: HMSO, 1906-1912, 1922-1937, 1976-2000.

Air Ministry / Ministry of Defence. Reports on the Health of the Royal Air Force [annual returns, 1920-1936, 1949, 1951-1963, 1965-1967, 1970-1976, 1981-2000]. London: HMSO, 1921-1937, 1950, 1952-1964, 1966-1968, 1971-1977, 1982-2001.

Merchant shipping:

Board of Trade / Ministry of Transport / Department of Trade and Industry etc. Casualties to vessels and accidents to men [annual returns, 1909-1911, 1913, 1914, 1919-1939, 1948-1988]. London: Board of Trade / Ministry of Transport / Department of Trade and Industry etc, 1910-1912, 1914, 1915, 1920-1940, 1949-1989.

Marine Accident Investigation Branch. Death inquiry files [1989-2020]. Southampton: Marine Accident Investigation Branch, 1990-2021.

Registry of Shipping and Seamen. Death inquiry files and death registers [1976-2010]. Cardiff: Registry of Shipping and Seamen.

Maritime and Coastguard Agency. Workforce surveys for UK merchant shipping [2006-2010]. Southampton: Maritime and Coastguard Agency.

General population:

Office for National Statistics. 20th and 21st Century mortality and population data.

2. Study years with missing suicide data, that were excluded from the analysis

General workforce	None
Royal Navy	1919, 1920, 1937-1939, 1946-1970, 1972-1974, 1978, 1979
Army	1900-1904, 1912-1914, 1919, 1920, 1937-1939, 1946-1974
Royal Air Force	(not formed until 1918), 1919, 1937-1939, 1946-1948, 1950, 1964, 1968, 1969, 1977-1980
Merchant shipping	1900-1908, 1912, 1946, 1947

3. International Classification of Diseases (ICD) codes for suicide

Time period	ICD version	ICD codes
1901–1910	ICD-1	190
1911–1920	ICD-2	155–163
1921–1930	ICD-3	165–174
1931–1939	ICD-4	163–171
1940–1949	ICD-5	163–164
1950–1957	ICD-6	E970–E979
1958–1967	ICD-7	E970–E979
1968–1978	ICD-8	E950–E959, E980–E989
1979–2000	ICD-9	E950–E959, E980–E989, excluding E988.8
2001–2020	ICD-10	X60–X84, Y10–Y34, excluding Y33.9 where coroner's verdict is pending
